# Deletion of D-Lactate Dehydrogenase A in *Neisseria meningitidis* Promotes Biofilm Formation Through Increased Autolysis and Extracellular DNA Release

**DOI:** 10.3389/fmicb.2019.00422

**Published:** 2019-03-05

**Authors:** Sara Sigurlásdóttir, Gabriela M. Wassing, Fanglei Zuo, Melanie Arts, Ann-Beth Jonsson

**Affiliations:** Department of Molecular Biosciences, The Wenner-Gren Institute, Stockholm University, Stockholm, Sweden

**Keywords:** *Neisseria meningitidis*, lactate dehydrogenase, eDNA, autolysis, biofilm

## Abstract

*Neisseria meningitidis* is a Gram-negative bacterium that asymptomatically colonizes the human nasopharyngeal mucosa. Pilus-mediated initial adherence of *N. meningitidis* to the epithelial mucosa is followed by the formation of three-dimensional aggregates, called microcolonies. Dispersal from microcolonies contributes to the transmission of *N. meningitidis* across the epithelial mucosa. We have recently discovered that environmental concentrations of host cell-derived lactate influences *N. meningitidis* microcolony dispersal. Here, we examined the ability of *N. meningitidis* mutants deficient in lactate metabolism to form biofilms. A lactate dehydrogenease A (*ldhA*) mutant had an increased level of biofilm formation. Deletion of *ldhA* increased the *N. meningitidis* cell surface hydrophobicity and aggregation. In this study, we used FAM20, which belongs to clonal complex ST-11 that forms biofilms independently of extracellular DNA (eDNA). However, treatment with DNase I abolished the increased biofilm formation and aggregation of the *ldhA*-deficient mutant, suggesting a critical role for eDNA. Compared to wild-type, the *ldhA*-deficient mutant exhibited an increased autolytic rate, with significant increases in the eDNA concentrations in the culture supernatants and in biofilms. Within the *ldhA* mutant biofilm, the transcription levels of the capsule, pilus, and bacterial lysis genes were downregulated, while *norB*, which is associated with anaerobic respiration, was upregulated. These findings suggest that the absence of *ldhA* in *N. meningitidis* promotes biofilm formation and aggregation through autolysis-mediated DNA release.

## Introduction

The Gram-negative human-restricted pathogen *Neisseria meningitidis* is the causative agent of meningitis and sepsis. Meningococci frequently colonize the respiratory epithelium as a non-invasive commensal, although in rare cases, the bacteria can cross the cell barrier and cause invasive disease (Pace and Pollard, 2012). The initial attachment of bacteria is facilitated by Type IV pili (Tfp), thin filaments that extend from the bacterial surface. After the initial adhesion of meningococci to the epithelium, the bacteria form Tfp-dependent three-dimensional aggregates, called microcolonies. Microcolony formation can develop into bacterial biofilms, i.e., bacterial communities that can persist and avoid removal by mechanical forces (Costerton et al., 1995). Studies have demonstrated the long-term asymptomatic carriage of *N. meningitidis* in the nasopharynx (Ala’Aldeen et al., 2000; Sim et al., 2000; Wilder-Smith et al., 2003) and the existence of microcolonies in patient biopsies (Harrison et al., 2002).

Several factors have been identified that contribute to meningococcal biofilm formation *in vitro*. In many bacterial species, the extracellular matrix is composed of polysaccharides, but *N. meningitidis* does not produce extracellular polysaccharides (Yi et al., 2004; Lappann et al., 2010). In most meningococcal genetic lineages, extracellular DNA (eDNA) is a major component of the extracellular matrix, contributing both to the initial binding to surfaces and the stabilization of the biofilm structure. The release of DNA by meningococci is thought to occur through bacterial lysis, mediated by lytic transglycosylases (MltA and MltB) and *N*-acetylmuramuyl-L-alanine amidase (AmpD) in early-stage biofilms. In late-stage biofilms, the outer membrane phospholipase A (OMPLA) mediates DNA release, which contributes to the resistance against mechanical forces (Lappann et al., 2010). Positively charged surface attached proteins are involved in biofilm formation by binding to eDNA. When surface exposed, *Neisseria* heparin-binding antigen (NhbA), as well as the autotransporters IgA protease and AutA can bind eDNA and promote biofilm formation. The autotransporter NalP can proteolytically cleave surface-bound NhbA and IgA protease, leading to reduced biofilm formation (Arenas et al., 2013, 2015). Biofilm formation in the absence of eDNA has also been reported, although this is relatively less protected against shear forces. eDNA-independent biofilms are usually formed by disease isolates and are therefore associated with high transmission rates (Lappann and Vogel, 2010; Lappann et al., 2010).

During biofilm formation, the availability of oxygen is limited (Werner et al., 2004; Barraud et al., 2006). It has been reported that the metabolism of *N. gonorrhoeae* adapts to growth in anaerobic environments via *aniA*, *ccp*, and *norB* upregulation (Falsetta et al., 2009). Meningococci also possess *aniA* and *norB.* Expression of *aniA* is under control of FNR (fumarate and nitrate reduction regulator), a master regulator of adaptation to anaerobic growth, and the two-component system NarQ/NarP in the presence of nitrite (Householder et al., 1999; Rock et al., 2005; Bartolini et al., 2006; Falsetta et al., 2009). Expression of *norB* is influenced by the nitric oxide concentrations (Householder et al., 2000; Rock et al., 2007). The meningococcal *aniA* may not always be active due to frame shift mutation (Barth et al., 2009), whereas *norB* is more conserved among strains. Further, the gonococcal *norB/aniA* cassette has been reported to by gene conversion appear in meningococcal strains causing urethritis (Tzeng et al., 2017). Deletion of *narP*, which encodes the regulator of the NarQ/NarP system, leads to defects in meningococcal biofilm formation (Jamet et al., 2013).

Unencapsulated strains are often isolated from healthy carriers, while for the development of disease, the capsule is almost indispensable (Stephens et al., 2007). Although the polysaccharide capsule is important for meningococcal immune evasion, it inhibits both intimate adhesion of the bacteria to host cells and biofilm formation on abiotic surfaces (de Vries et al., 1996; Yi et al., 2004; Lappann et al., 2006). Downregulation of the capsule is thought to occur upon Tfp-dependent adhesion to host cells and during biofilm formation. The absence of the capsule will expose surface-expressed molecules that support intimate adhesion, invasion, and biofilm modes of growth (Deghmane et al., 2002; Tzeng et al., 2008; O’Dwyer et al., 2009). It has, however, been reported that meningococcal biofilm formation on human airway epithelial cells is not inhibited by the presence of capsule (Neil et al., 2009). Lipooligosaccharide (LOS) has also been shown to have negative effects on meningococcal biofilm formation (Yi et al., 2004; Lappann and Vogel, 2010). A decrease in LOS biosynthesis transcripts has been observed during biofilm growth (O’Dwyer et al., 2009). Tfp is important in the initial attachment of meningococci to host cell surfaces, for bacterial aggregation, and twitching motility (reviewed in Craig et al., 2004). Tfp-mediated bacterial aggregation has been shown to play a role in meningococcal biofilm formation. Additionally, non-pilated and non-aggregative strains might form biofilms, although their flat architectures are different from those of microcolony-based biofilms (Yi et al., 2004; Lappann et al., 2006). It has been proposed that the reduction in twitching motility, rather than aggregation, in these strains affects the establishment of microcolonies within the biofilms (Wolfgang et al., 1998; Lappann et al., 2006). The surface-exposed autotransporter AutA has been reported to promote both aggregation and biofilm formation by binding to itself and eDNA (Arenas et al., 2015).

We have previously found that host-cell derived lactate induces the dispersal of meningococcal aggregates in liquid cultures (Sigurlasdottir et al., 2017). Additionally, lactate dehydrogenases (LDHs) have been implicated in the biofilm formation of multiple bacteria. In iron-supplemented medium, LDH promotes biofilm growth of the gut bacteria *Enterococcus faecalis* (Keogh et al., 2018). LDH promotes biofilm formation under anaerobic conditions of the oral bacteria *Streptococcus sanguinis* (Ge et al., 2016). Conversely, in *Escherichia coli*, deletion of *ldhA* and other genes that are important in acetate metabolism increased the level of biofilm formation. The loss of *ldhA* and lactate dehydrogenase regulator (*ldhR*) in *Burkholderia cepacia* increased cell viability and production of extracellular matrix, while aggregation and biofilm formation were reduced (Silva et al., 2017). However, the role of the enzymes involved in lactate metabolism in biofilm formation of *N. meningitidis* is not clearly understood. We, therefore, investigated the roles of the enzymes involved in lactate metabolism in meningococcal biofilm formation.

In the present study, we demonstrate that the absence of D-LDH LdhA increased the amount of biofilm formed by the meningococcal strain FAM20. Additionally, *ldhA* mutants showed stronger cell surface hydrophobicity and aggregation. The observed increased biofilm formation and aggregation were the results of the increase in the autolytic rate and the amount of eDNA. Taken together, our findings show that the deletion of *ldhA* promotes biofilm formation and aggregation through autolysis-mediated DNA release.

## Materials and Methods

### Bacterial Strains and Growth Conditions

*Neisseria meningitidis* strain FAM20 (FAM18 derivative) and its mutants deficient in capsule operon (Δ*cap*) or lactate metabolic genes (Δ*lctP*, Δ*lldA*, Δ*ldhD*, and Δ*ldhA*) have been described previously (Jones et al., 2009; Sigurlasdottir et al., 2017). Bacterial strains were grown on GC agar plates containing a 1% Kellogg’s supplement for 16–18 h at 37°C and 5% CO_2_. GC liquid containing 1% Kellogg’s supplement was used for experiments performed in liquid culture. For the selection of FAM20 mutant strains, appropriate antibiotics were used: tetracycline (1 μg/ml), chloramphenicol (2 μg/ml), and kanamycin (50 μg/ml). DMEM containing 10% FBS was used as a medium for human cells.

### Construction of Mutant Strains

Phusion DNA polymerase (Thermo Fisher Scientific) was used for PCR amplification. All primers used in the study are presented in Table 1. All constructs generated were incorporated into the genome of wild-type *N. meningitidis* FAM20 or relevant mutants by performing spot transformation, plated and selected on plates containing relevant antibiotics. PCR and sequencing were performed to confirm the correct location and sequence of the constructs in the genome.

**Table 1:** Primers used for generation of mutants.

Primer	Sequence (5′– 3′)	Reference
DUS_UHS_fw	*ATGCCGTCTGAA*AATTAAGTTAGAATTATCCCTAT	This work
UHS_ldhA_rev	TTTCCATTTCAAAACAAATCCAAAATCATACTGCCATAATT	This work
ldhA_Cm_DHS_fwd	CCAACTTGGCAACATGCCGTCTGAATGAGACGTTGAT	This work
DHS_rev	TTGCTAACAGAAAACTCTACTCC	This work
UHS_ldhA_fwd	ATGATTTTGGATTTGTTTTGAAATGGAAATGCCGTGCA	This work
ldhA_Cm_DHS_rev	CATTCAGACGGCATGTTGCCAAGTTGGAAGTGA	This work
ldhA_up_fwd	*ATGCCGTCTGAA*TTCAGTGTATTATGCCGT	Sigurlasdottir et al., 2017
ldhA_dn_rev	TAAAACACGTCAGCCGTCG	Sigurlasdottir et al., 2017

*The DNA uptake sequence is marked in italic. The overlapping sequences for fusion PCR are underlined.*

To generate the construct for complementation of the Δ*ldhA* mutation, a fusion PCR was performed. The *ldhA* gene, including its native promoter, was introduced into a non-coding region between NMC0075 and NMC0080. First, an upstream homologous sequence containing the DUS sequence was amplified from FAM20 genomic DNA using primers DUS_UHS_fw and UHS_ldhA_rev. The downstream homologous sequence containing a chloramphenicol resistance cassette was amplified from a plasmid pDONR P4-P1R, as previously described (Kuwae et al., 2011), with primers ldhA_Cm_DHS_fwd and DHS_rev. The *ldhA* gene and its native promoter were amplified from FAM20 genomic DNA using primers UHS_ldhA_fwd and ldhA_Cm_DHS_rev. After amplification of all PCR products containing overlapping sequences, two-step fusion PCR reactions were performed. The first reaction was performed in the absence of primers to anneal overlapping sequences of the non-coding regions to *ldhA* and its native promoter. Later, the primers DUS_UHS_fw and DHS_rev were added to amplify the construct, which was then integrated into the FAM20 Δ*ldhA* genome.

To obtain a Δ*cap*/Δ*ldhA* double mutant, the Δ*ldhA* construct was amplified with the primer pair ldhA_up_fwd and ldhA_dn_rev (Sigurlasdottir et al., 2017). The complete construct was then integrated into the Δ*cap* mutant.

### Biofilm Assay Under Static Conditions

The assay has been described previously (Engman et al., 2016). Bacteria were grown on GC plates overnight and suspended to an OD of 0.05. One-hundred microliters of the bacterial solution was added in triplicate to 96-well polystyrene plates and allowed to grow under static conditions for 24 h at 37°C and 5% CO_2_. After incubation, the samples were washed twice with PBS to remove unbound bacteria. For quantification, the biofilm was stained for 2 min with 0.3% crystal violet, washed twice with PBS to remove unbound dye and solubilized in 30% acetic acid. The absorbance was measured at 630 nm (Yi et al., 2004) using a POLARstar Omega microplate reader. When necessary, DNase I was added at a final concentration of 100 μg/ml. As a control, DNase I was heat inactivated for 10 min at 65°C. Biofilm assays were also performed in the presence of 0.1, 1, and 10 mM sodium acetate. The assay was performed at least three times using triplicate samples. The absorbance values were normalized against the wild-type.

### Growth Assays

The wild-type strain and its isogenic mutants were resuspended to an OD_600_ of 0.1. Cultures were grown under shaking conditions at 37°C and 5% CO_2_. The absorbance was measured every hour for 7 h. The growth assay was performed three times in duplicate. For OD measurement after 24 h, the wild-type and Δ*ldhA* strains were resuspended to an OD of 0.05, and the cultures were grown under shaking conditions at 37°C and 5% CO_2_. Samples were measured three times in duplicate. For OD measurement in the presence of DNase I, the wild-type and Δ*ldhA* strains were resuspended to an OD of 0.05 with or without 100 μg/ml DNase I and grown under shaking conditions at 37°C and 5% CO_2_. Samples were measured twice.

### Microscopy

The wild-type, Δ*ldhA*, and Δ*ldhA/ldhA* bacteria were resuspended to an OD_600_ of 0.05 and one ml was seeded per well containing a glass cover slip. After 24 h of incubation the wells were washed twice with PBS and fixed with 2% formaldehyde/PBS for 30 min. The samples were stained with 330 nM DAPI/PBS for 5 min, washed three times with PBS, and mounted with Vectashield mounting medium (Vector Laboratories). The samples were examined under Zeiss LSM 780 confocal laser scanning microscope. Image stacks at 0.4 μm intervals were acquired using 63×/1.4 NA oil objective and processed using ZEN Black Zeiss software. Nine stacks were combined to create each image.

### Quantification of Bacterial Aggregation

The sedimentation assay has been described previously (Helaine et al., 2005). Bacterial suspensions were filtered through 5 μm filters to remove aggregates, and the absorbance was adjusted to OD_600_ of 0.1. After 3 h of incubation at 37°C and 5% CO_2_ with agitation, the cultures were moved to static conditions at room temperature. Sedimentation of bacterial aggregates was examined by measuring the OD_600_ at 20 min intervals. During DNase I treatment, a final concentration of 100 μg/ml was used. The assay was performed three times in duplicate and single samples in the presence of DNase. The absorbance values were normalized against the first time point (0 min). The rate of sedimentation was calculated for the first 20 min using the following formula (OD_600_20 min - OD_600_0 min)/(20 min – 0 min). The result gave the decline in absorbance per minute (Abs/min).

### Western Blot

Bacteria from plates were resuspended to an OD of 0.05 and incubated for 3 h at 37°C and 5% CO_2_ under shaking conditions. Samples were taken, centrifuged and resuspended in 1 × sample buffer containing β-mercaptoethanol. The samples were heated at 95°C for 5 min. Proteins were separated on a gradient gel (4–15%, Bio-Rad) and transferred to an Immobilon-P membrane. For detection of PilE, a rabbit polyclonal antibody (1:5000) (Sjolinder and Jonsson, 2007) was used as a primary antibody, and IR-reactive dye conjugated rabbit antibody was used as a secondary antibody. The membrane was stripped to remove antibodies, and EF-Tu monoclonal mouse antibody (Hycult Biotech) was used together with IR-reactive dye conjugated anti-mouse antibody as a loading control. Blots were imaged with Odyssey IR scanner at 700 and 800 nm. For quantification, Image J (version 1.48) was used.

### Adherence Assays

The human epithelial cell line FaDu (ATCC HTB-43) was grown to 100% confluence in 24-well plates. Wild-type, Δ*ldhA*, and Δ*ldhA/ldhA* bacteria were resuspended in DMEM supplemented with 1% FBS, filtered through a 5 μm pore filter to break bacterial aggregates, and used to infect cells at an MOI of 100 for 3 h at 37°C in a 5% CO_2_ environment. Unbound bacteria were washed away three times. The cells were lysed using 1% saponin, and the adhered bacteria were quantified by plating serial dilutions on GC agar plates. Cells were maintained in Dulbecco’s modified Eagle’s medium containing GlutaMAX and pyruvate (DMEM; Thermo Fisher Scientific) and supplemented with 10% heat-inactivated fetal bovine serum (FBS; Sigma-Aldrich).

### Microbial Adhesion to Solvents (MATS)

This assay has been described previously (Ly et al., 2006). Briefly, bacteria from plates grown overnight were resuspended in PBS and filtered through 5 μm filters to remove aggregates. The absorbance of the filtered bacterial solution was adjusted to an OD_600_ of 0.4 and mixed with hexadecane at a ratio of 4:1. To mix the two phases, the solution was vortexed for 30 s and then allowed to stand at room temperature to separate for 15 min. The absorbance of the aqueous phase was measured, and the percentage of hydrophobicity was calculated using the formula [1 – (OD_600_2/OD_600_1)] × 100%. The assay was performed three times using triplicate samples.

### ELISA

The capsule level was quantified using ELISA. Bacteria were grown overnight on GC plates and then resuspended in PBS to OD_600_ of 0.1. Bacterial solutions were heat-inactivated at 56°C for 60 min. The ELISA plate was coated with 100 μl of the bacterial solutions overnight at 4°C and then blocked in 2% bovine serum albumin (BSA) for 2 h. Wells were incubated with 95/678 monoclonal anti-capsule antibody (Jones et al., 2009) diluted 1:400 in 2% BSA for 2 h and then horseradish peroxidase-conjugated goat anti-mouse immunoglobulin G (diluted 1:5000 in 2% BSA) for 1 h. 3,3′,5,5′-tetramethylbenzidine (TMB) was used to detect peroxidase bound to the plate, and 1 M HCl was used as a stop solution. The absorbance was measured at 450 nm (reference wavelength, 490 nm) using a Spectramax i3x microplate reader. The assay was performed three times using triplicate samples. The absorbance values were normalized against those of the wild-type.

### Autolysis Under Non-growth Conditions

The assay was adapted from a previously described method (Garcia and Dillard, 2006). Wild-type and Δ*ldhA* bacteria from plates were resuspended to an OD_600_ of 0.05 and grown for 3 h. The bacteria were centrifuged, washed, resuspended in PBS and diluted to OD_600_ of 0.1 in 30 mM Tris-HCl buffer pH 8.0 or 30 mM Tris-HCl buffer pH 6.0. The decrease in turbidity was measured every 10 min at room temperature for 1 h and after that every 20 min. The initial turbidity (%) was calculated by using the formula (OD_600_ × min/OD_600_ 0 min) × 100%. The bacteria were resuspended before every measurement to prevent the effects of sedimentation influencing the assay. The assay was performed four times.

### DNA Quantification in Culture Supernatants and Biofilms

The assay has been described previously (Hamilton et al., 2005; Tang et al., 2013). Briefly, bacteria were resuspended to an OD_600_ of 0.05 and grown for 3 h. The bacteria were centrifuged at 13,000 rpm for 3 min, and the supernatants were collected and incubated with Quant-iT PicoGreen dsDNA dye (Thermo Fisher Scientific) at a 1:1 ratio. To determine the eDNA concentration in biofilms, a PicoGreen solution was prepared in TE buffer (1 μl:199 μl) and added directly to biofilms that had been washed two times with PBS. The solution was mixed in the well by pipetting 10 times. Samples mixed with the dye were incubated for 2–5 min before measurements. The dye has been shown to detect both single-stranded and double-stranded DNA (Hamilton et al., 2005; Lappann et al., 2010). DNA was quantified based on a Lambda DNA standard. The fluorescence was measured using the excitation and emission at 485 and 535 nm using a Spectramax i3x microplate reader.

### Quantitative Real-Time PCR Analysis

For comparison of gene expression of the wild-type and Δ*ldhA* strains, biofilm assays were performed as indicated above in 24-well glass-bottom plates in 1 ml and incubated for 24 h. Wells were washed carefully two times with PBS, and then the biofilm was resuspended in RNA-protect Bacteria Reagent (Qiagen) diluted at a 1:2 ratio. The samples were mixed extensively and incubated for 5 min. DNase I (final concentration at 0.5 mg/ml) and proteinase K (final concentration at 20 μg/ml) were added to the samples and incubated for 15 min to disrupt the biofilm. For log phase bacterial cultures, the wild-type and Δ*ldhA* bacteria were resuspended to an OD_600_ of 0.05 and grown under shaking conditions for 3 h; then, samples were collected. Samples were resuspended in RNA-protect Bacteria Reagent (Qiagen) diluted at a 1:2 ratio, vortexed, and incubated for 5 min.

For RNA isolation of both log phase and biofilm samples, the bacteria were pelleted by centrifugation for 1 min at 15,000 × *g*, and RNA was purified using the RNeasy plus mini kit (Qiagen) according to the manufacturer’s protocol. The RNA yield and quality were analyzed using NanoDrop 8000. SuperScript VILO Master Mix (Thermo Fisher Scientific) with random hexamers was used for cDNA synthesis. LightCycler 480 Real-Time 480 SYBR Green I Master mix (Roche) was used to amplify the resulting cDNA in a LightCycler 480 Real-Time PCR System. The housekeeping gene *rpsJ* was used as a reference. The PCR program was according to the manufacturer’s instructions, with 40 cycles of amplification and annealing temperatures of 55 or 60°C. Melting curves were analyzed to verify primer pair specificity. Relative expression was analyzed using the LightCycler 480 Real-Time PCR System software. All primers used for the qPCR analysis are listed in Table 2.

**Table 2:** qPCR primers used in the study.

Primer	Sequence (5′– 3′)	Reference
aniA_qPCR_fw	AGGCGAAACCGTGCGTATGT	This work
aniA_qPCR_rev	GGAAGACACTAGGTTCGGAC	This work
narP_qPCR_fw	GACCGCCAAACTCGTTAAAAG	This work
narP_qPCR_rev	GAGATAGCCCAAGATTTCCAG	This work
siaD_qPCR_fw	CCTACTACCCAATGTCTGTCAA	This work
siaD_qPCR_rev	GCTCTTCAATTAAAGCGGTGTTC	This work
pilE_qPCR_fw	TATTCCGACAACGGCACATTCCC	Kuwae et al., 2011
pilE_qPCR_rev	CCTTCAACCTTAACCGATGCCA	Kuwae et al., 2011
pilX_qPCR_fw	CGGGGACGGGTTATACTTT	Sigurlasdottir et al., 2017
pilX_qPCR_rev	GGCATCACGGCATTTGTATC	Sigurlasdottir et al., 2017
nalP_qPCR_fw	AGTCTCGCCGCTACCGTCTAT	This work
nalP_qPCR_rev	CCACTTTCAGCAGTTTGCCCA	This work
nhbA_qPCR_fw	AGATGCCGCTGATTCCCGTCAA	This work
nhbA_qPCR_rev	TTTTCCGCCCCGTAAGTCAGA	This work
mltA_qPCR_fw	CGAGCATCCGTATGTTTCCATC	This work
mltA_qPCR_rev	ATAAGACTTAATGCCCTGCATGG	This work
mltB_qPCR_fw	ATTATGACGGGGACGGACATC	This work
mltB_qPCR_rev	GCCAATGATTGCCTGAACAT	This work
ampD_qPCR_fw	CTTCATTCGGCGGCAGGGAAAA	This work
ampD_qPCR_rev	CAGATTGCGTCCAACAAGGCT	This work
rpsJ_qPCR_fwd	TTGGAAATCCGCACCCACTT	Kuwae et al., 2011
rpsJ_qPCR_rev	TACATCAACACCGGCCGACAAA	Kuwae et al., 2011

### Acetate Quantification

Culture medium was collected from 24 h old biofilms and centrifuged at 13,000 rpm for 3 min, and the supernatants were collected. The total acetate concentration was measured using Acetate Colorimetric Assay Kit (Sigma-Aldrich) according to manufacturer’s instructions. As recommended by the manufacturer, a sample blank, excluding the Acetate Enzyme mix, was set up for each sample to adjust the effects of ATP and NADH background.

### Statistical Analysis

Two-tailed, unpaired Student’s *t*-tests were used when comparing between two groups. ANOVA with Bonferroni’s *post hoc* test was used when comparing differences between more than two groups. *P*-values below 0.05 were accepted as statistically significant.

## Results

### Deletion of LdhA Influences the Level of Meningococcal Biofilm Formation Under Static Conditions

**FIGURE 1:**
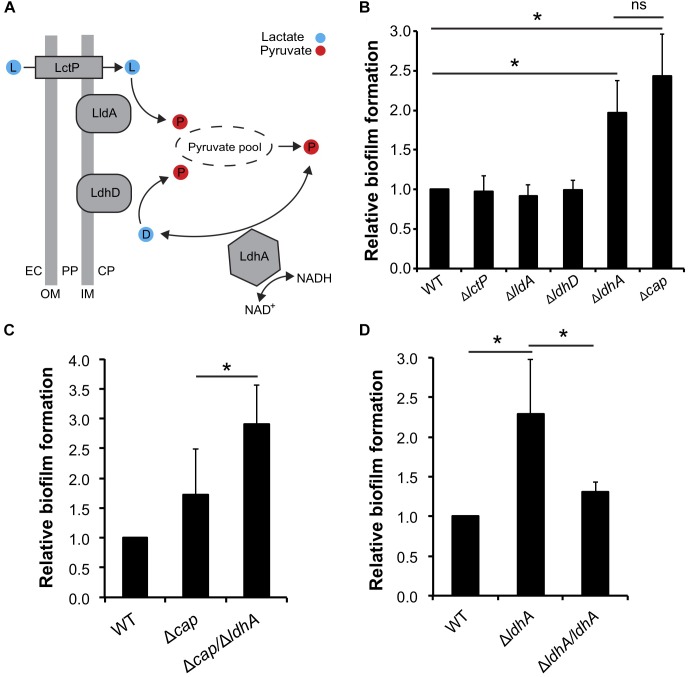
Deletion of *ldhA* promotes meningococcal biofilm formation *in vitro*. **(A)** Predicted role of lactate dehydrogenases (LDHs) in *N. meningitidis*, adapted from Atack et al. (2014). Abbreviations: LctP, lactate permease; LldA, respiratory L-LDH; LdhD, respiratory D-LDH; LdhA, cytoplasmic NAD^+^ dependent D-LDH; NAD^+^, oxidized nicotinamide adenine dinucleotide; NADH, reduced nicotinamide adenine dinucleotide; EC, extracellular space; PP, periplasm; CP, cytoplasm; OM, outer membrane; IM, inner membrane. **(B)** Biofilm formation by the wild-type, Δ*lctP*, Δ*lldA*, Δ*ldhD*, and Δ*ldhA* mutant strains. Δ*cap* was used as a positive control. **(C)** Biofilm formation by the wild-type, Δ*cap*, and Δ*capA/*Δ*ldhA* strains. **(D)** Biofilm formation by the wild-type, Δ*ldhA*, and Δ*ldhA/ldhA* strains. For all biofilm experiments, bacteria were resuspended in GC liquid supplemented with 1% Kellogg’s supplement to an OD_600_ of 0.05 and grown under static conditions at 37°C and 5% CO_2_ for 24 h. The biofilm was washed twice in PBS and then stained with crystal violet. After washing two times with PBS, the biofilm was dissolved with acetic acid and quantified by measuring the absorbance at 630 nm. Experiments were performed at least three times in triplicate. The bars represent the means, with error bars representing the standard deviations. ^∗^*p* < 0.05. ns, non-significant.

**FIGURE 2:**
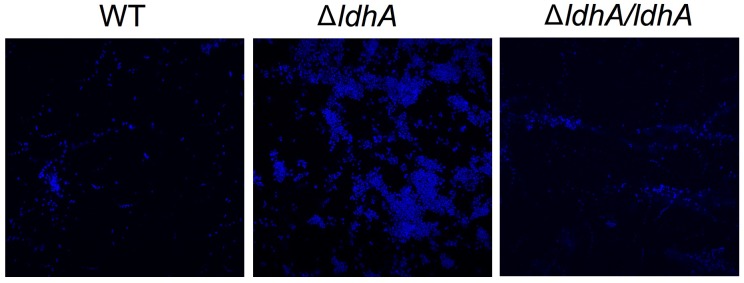
Wild-type, Δ*ldhA*, and Δ*ldhA/ldhA* biofilms visualized by confocal microscopy. Wild-type, Δ*ldhA*, and Δ*ldhA/ldhA* bacteria were resuspended to OD_600_ of 0.05 and grown under static conditions at 37°C and 5% CO_2_ for 24 h on glass coverslips. After washing two times with PBS the samples were fixed with 2% formaldehyde and stained with DAPI.

We have previously shown that lactate induces the dispersal of meningococcal aggregates (Sigurlasdottir et al., 2017). Because aggregation can lead to the establishment of biofilms, we aimed to examine whether deficiencies in lactate metabolism influenced *N. meningitidis* biofilm formation. Lactate permease (LctP) is responsible for lactate uptake (Exley et al., 2005, 2007), and lactate dehydrogenases (LDHs) catalyze the conversion of lactate into pyruvate. Meningococci are known to contain at least three LDHs (Fischer et al., 1994). LdhA is an NAD^+^-dependent cytoplasmic D-LDH that catalyzes the reversible conversion of pyruvate into D-lactate. Membrane-bound LdhD (D-LDH) and LldA (L-LDH) are respiratory enzymes that are restricted to the conversion of lactate to pyruvate (Figure 1A; Atack et al., 2014). As the capsule has been shown to have a negative impact on meningococcal biofilm formation on abiotic surface, we used an unencapsulated mutant of *N. meningitidis* FAM20 as the control (Jones et al., 2009). Analysis of biofilm levels of the four isogenic mutants lacking the genes encoding the three LDHs and LctP showed that Δ*ldhA* formed more biofilm compared to the wild-type. Deletion of Δ*lctP*,Δ*lldA*, and Δ*ldhD* did not have any effect on biofilm formation (Figure 1B). We also detected no difference in the growth rate between the mutants and wild-type strains in the medium used (Supplementary Figures 1A,B). Because meningococcal biofilm assays are usually performed in capsule-deficient backgrounds, we constructed a Δ*cap/*Δ*ldhA* double-mutant strain. The double-mutant strain had increased biofilm formation compared to that of Δ*cap* (Figure 1C), further supporting the role of *ldhA* in biofilm formation. We next constructed a *ldhA*-complemented strain (Δ*ldhA/ldhA*) with a copy of *ldhA* containing its native promoter into a non-coding region in the chromosome. The biofilm formation of Δ*ldhA/ldhA* was at a similar level to that of the wild-type strain (Figure 1D). The increase in biofilm formation by the Δ*ldhA* compared to that of the wild-type and Δ*ldhA/ldhA* strains was also detected using confocal microscopy (Figure 2). Taken together, these data suggest that deletion of the lactate dehydrogenase *ldhA* promotes biofilm formation.

### Deletion of *ldhA* Increases Meningococcal Aggregation and Cell Surface Hydrophobicity

**FIGURE 3:**
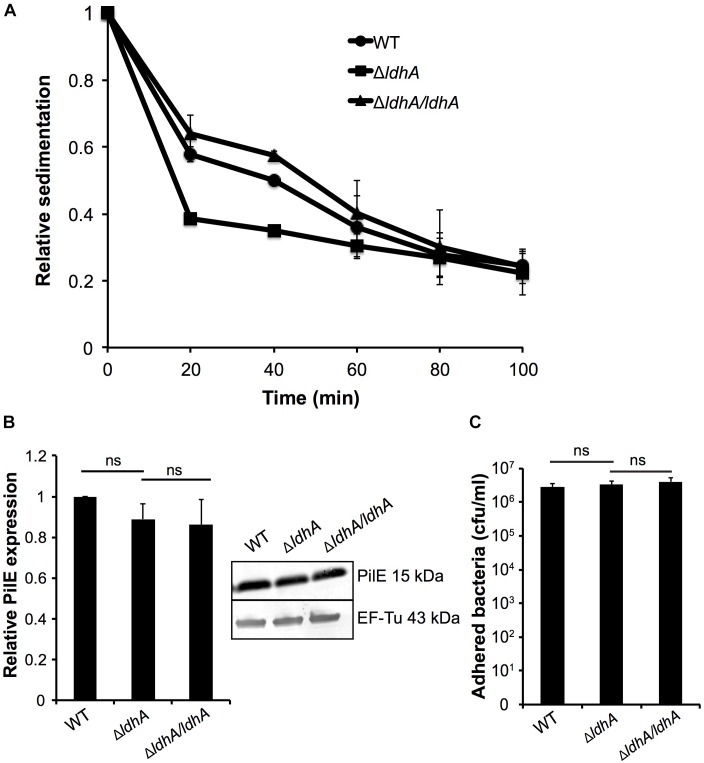
Deletion of *ldhA* increases meningococcal aggregation. **(A)** Bacterial aggregation of the wild-type, Δ*ldhA*, and Δ*ldhA/ldhA* strains was examined by the sedimentation assay. Bacteria were grown for 3 h to log phase under shaking conditions at 37°C and 5% CO_2_ and then moved to static conditions at room temperature. The absorbance (OD_600_) of the top layer of the culture was measured every 20 min. Data are presented as relative values compared to the 0 min time point (set to 1) for all strains. **(B)** Western blot analysis and quantification of PilE expression in the wild-type, Δ*ldhA*, and Δ*ldhA/ldhA* strains after 3 h of growth in liquid cultures. After PilE detection, the membrane was stripped, and the level of EF-Tu was examined as a loading control. Data are represented as relative values, and the PilE expression of the wild-type is set to 1. **(C)** Quantification of the adherence of the wild-type, Δ*ldhA*, and Δ*ldhA/ldhA* strains to the epithelial cell line FaDu. Cells at 100% confluence were infected at an MOI of 100 and incubated for 3 h. All unbound bacteria were removed by washing. Bound bacteria were released by a saponin treatment and quantified by viable counting on GC plates. Experiments were performed at least three times in triplicate. The bars represent the means with error bars representing the standard deviations. ns, non-significant.

Meningococcal aggregation has been shown to strongly influence biofilm architecture (Yi et al., 2004; Lappann et al., 2006; Arenas et al., 2015). We, therefore, examined whether deletion of *ldhA* had any effect on bacterial aggregation using a sedimentation assay. Bacteria were grown for 3 h under shaking and were then transferred to static conditions; the absorbance at OD_600_ of the surface layer was measured every 20 min. The Δ*ldhA* sedimented faster compared to the wild-type and Δ*ldhA/ldhA* strains (Figure 3A). The rate of decline in absorbance was calculated for the first 20 min. The Δ*ldhA* (-0.031 Abs/min) had a significant increase (*p* < 0.05) in rate compared to that of the wild-type (-0.021 Abs/min) and Δ*ldhA/ldhA* (-0.018 Abs/min) strains. Because pilus expression influences meningococcal aggregation, we performed Western blot analysis to quantify the amount of the major pilus subunit PilE. We did not observe any differences in PilE expression between the wild-type and Δ*ldhA* strains (Figure 3B and Supplementary Figure 3). Also, we did not observe any difference in the level of adhesion to human pharyngeal epithelial FaDu cells for Δ*ldhA* compared to that of the wild-type and Δ*ldhA/ldhA* strains (Figure 3C).

**FIGURE 4:**
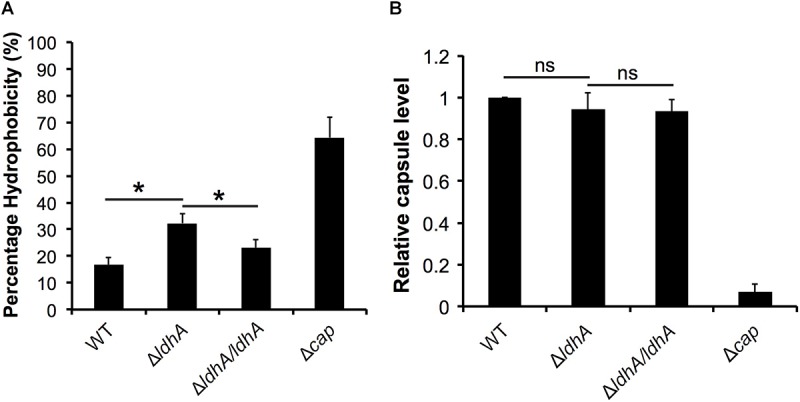
Surface hydrophobicity is increased in the Δ*ldhA*, but the capsule level remains constant. **(A)** A microbial adhesion to solvents (MATS) assay was used to measure the surface hydrophobicity of the wild-type, Δ*ldhA*, and Δ*ldhA/ldhA* strains. The Δ*cap* mutant was used as a control. Bacteria were resuspended in PBS to an OD of 0.4, mixed with hexadecane at a 1:4 ratio and vortexed extensively. After a 15 min incubation, the absorbance (OD_600_) of the aqueous phase was measured. **(B)** The level of the polysaccharide capsule was detected with anti-serogroup C capsule antibodies using an indirect ELISA. The plate was coated with a heat-killed bacterial suspension. The absorbance values were normalized against those of the wild-type strain. Experiments were performed at least three times in triplicate. The bars represent the means, with error bars representing the standard deviations. ^∗^*p* < 0.05. ns, non-significant.

As there is a correlation between biofilm formation and hydrophobicity (Yi et al., 2004), we next examined the surface hydrophobicity. We observed elevated cell surface hydrophobicity in Δ*ldhA* compared to wild-type and Δ*ldhA/ldhA* strains (Figure 4A). The capsule-negative mutant (Δ*cap*) was more hydrophobic than the other strains (Figure 4A). To exclude the possibility that the increased Δ*ldhA* surface hydrophobicity was due to reduced capsule expression, we quantified the capsule level. Capsule ELISA revealed no differences in the capsule produced by the Δ*ldhA* mutant compared to that produced by the wild-type strain (Figure 4B). These results indicate that Δ*ldhA* has stronger bacterial aggregation and surface hydrophobicity than the wild-type strain while there was no difference in the level of adhesion to host cells, PilE, and capsule.

### Increased Aggregation and Biofilm of Δ*ldhA* Is Dependent on Extracellular DNA

**FIGURE 5:**
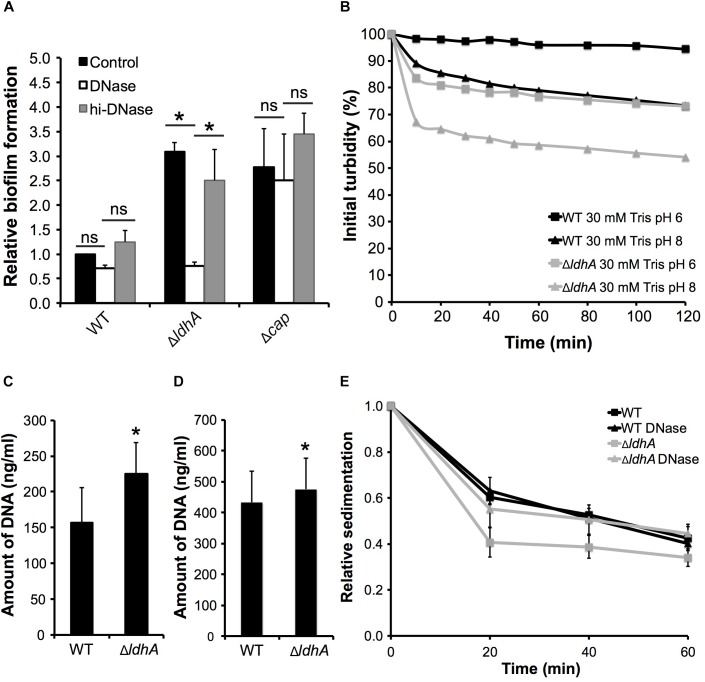
Increased biofilm formation and aggregation by Δ*ldhA* is associated with an increase in extracellular DNA and autolysis. **(A)** Biofilm formation by the wild-type, Δ*ldhA*, and Δ*cap* mutant strains in the presence or absence of DNase I. Δ*cap* was used as a positive control. Bacteria were resuspended in GC liquid supplemented with 1% Kellogg’s with or without DNase I to OD_600_ of 0.05 and grown under static conditions for 24 h. Heat-treated DNase I was used as control (hi–DNase). Washed biofilms were stained with crystal violet and dissolved with acetic acid. The absorbance was measured at 630 nm. The experiment was performed three times in triplicate. **(B)** Autolysis under non-growth conditions. The wild-type and the Δ*ldhA* strains were resuspended in GC liquid supplemented with 1% Kellogg’s, to an OD_600_ of 0.05 and grown for 3 h. Bacteria were centrifuged, washed with PBS, and resuspended in 30 mM Tris-HCL buffers at pH 6 or 8 to an OD_600_ of 1. Absorbance values were acquired every 10 min for the first hour and at 20 min intervals during the second hour. The values were used to calculate the percentage of initial turbidity. One representative experiment, performed in duplicate, out of four is shown. Detection of eDNA in the culture supernatants **(C)** and in biofilms **(D)** of the wild-type and Δ*ldhA* strains using a fluorescence-based Quant-IT PicoGreen dsDNA assay kit. Experiments were performed three times in triplicate. **(E)** Sedimentation of wild-type and Δ*ldhA* aggregates grown in the presence or absence of DNase I. Bacteria were grown for 3 h in the presence or absence of DNase I under shaking conditions at 37°C and 5% CO_2_ and then moved to static conditions at room temperature. The absorbance (OD_600_) of the top layer of the culture was measured every 20 min. Data are presented as relative values compared to the 0 min time point (set to 1). Experiments were performed three times. Unless stated, the bars represent the means, with error bars representing the standard deviations. ^∗^*p* < 0.05. ns, non-significant.

Since eDNA is often a major component of the meningococcal extracellular matrix (Lappann et al., 2010) and has been shown to affect bacterial surface hydrophobicity in for example streptococci (Das et al., 2010), we examined whether the increased biofilm formation of Δ*ldhA* was dependent on eDNA. DNase I was added to bacterial solutions before incubation under static conditions for 24 h. The wild-type FAM20 belongs to clonal complex ST-11 strains, which are known to form eDNA-independent biofilms (Lappann et al., 2010). DNase I treatment of the wild-type and Δ*cap* mutant strains did not change the level of biofilm formation (Figure 4A). However, the presence of DNase I significantly (*p* < 0.05) reduced the biofilm formation of Δ*ldhA* compared to that of those untreated or incubated with heat-inactivated DNase I (Figure 5A). We further show that treatment with DNase did not affect the growth of the wild-type and Δ*ldhA* strains (Supplementary Figure 2). These data indicate that eDNA plays a role in the increased biofilm formation of the Δ*ldhA* mutant.

Because meningococcal eDNA release occurs via cell lysis (Lappann et al., 2010), we next examined autolysis of the Δ*ldhA* and wild-type strains in Tris buffer at pH 6.0 or 8.0. Tris buffer pH at 8.0, but not at pH 6.0, has previously been shown to lead to autolysis in *N. gonorrhoeae* (Bos et al., 2005; Garcia and Dillard, 2006). Bacteria were resuspended in the buffers, and lysis was quantified by measuring the absorbance every 10 min for 1 h and after that at 20 min intervals. Bacteria were resuspended before every OD measurement to prevent the effects of sedimentation. We detected increased autolysis of Δ*ldhA* compared to that of the wild-type strain at pH 8.0. While Tris buffer at pH 6.0 did not induce autolysis in the wild-type strain, the Δ*ldhA* exhibited autolysis in the pH 6.0 buffer (Figure 5B). To test whether there was a difference in DNA release, we quantified the eDNA from culture supernatants after 3 h of growth. The amount of eDNA measured was higher in Δ*ldhA* culture supernatants and in biofilms compared to that in the wild-type culture supernatants (Figure 5C,D).

These results suggest that an increase in eDNA release mediated by autolysis is the reason for the increased bacterial aggregation and biofilm formation. We, therefore, performed sedimentation assays in the presence or absence of DNase I. We found that the DNase I treatment abolished the enhanced aggregation in Δ*ldhA*, while it did not have an effect on the wild-type strain (Figure 5E). The rate of sedimentation was calculated for the first 20 min. The Δ*ldhA* (-0.030 Abs/min) had a significant (*p* < 0.05) increase in sedimentation rate compared to that of the wild-type (-0.020 Abs/min) and Δ*ldhA* treated with DNase (-0.022 Abs/min). To summarize, these results indicate that an increase in eDNA release, mediated by autolysis, is the reason for the enhanced aggregation and biofilm formation in Δ*ldhA* mutant.

### Differential Expression of Biofilm-Related Genes in Wild-Type and Δ*ldhA N. meningitidis*

**FIGURE 6:**
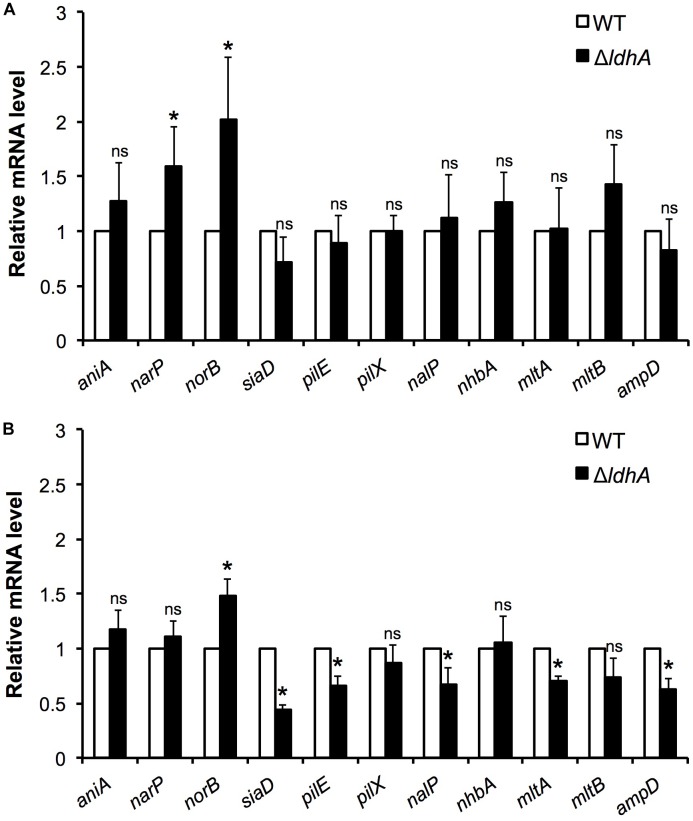
Comparison of gene expression in log phase cultures and biofilms. Gene expression in wild-type and Δ*ldhA* bacterial cultures **(A)** and biofilms **(B)**. RNA was isolated from bacteria grown to log phase for 3 h and from 24 h old biofilms to generate cDNA to use as a template for qPCR analysis. The gene encoding the ribosomal protein RpsJ was used as a housekeeping gene for data normalization of target mRNA. Experiments were performed four times. The bars represent the means, with error bars representing the standard deviation. ^∗^*p* < 0.05. ns, non-significant.

To explore whether the deletion of *ldhA* influences the expression of genes previously linked to biofilm formation, we performed qPCR analyses. Total RNA was isolated from wild-type and Δ*ldhA* mutant harvested from both 3 h log phase shaking cultures and 24 h biofilms. We detected changes in the expression of genes involved in anaerobic respiration. Expression of *norB* was increased in the Δ*ldhA* mutant both during log phase and biofilm growth. Although the expression of *narP* was significantly(*p* < 0.05) induced during log phase, we did not detect within-biofilm changes in the Δ*ldhA* mutant (Figure 6A,B). No significant changes in the expression of *aniA* were detected (Figure 6A,B).

Consistent with the capsule ELISA and PilE detection assay results, we did not find any changes in *siaD* and *pilE* expression in log cultures (Figure 6A). However, *pilE* and *siaD* were downregulated upon biofilm formation in the Δ*ldhA* mutant (Figure 6B). The expression of *nalP* and lysis-associated *mltA* and *ampD* was downregulated in the Δ*ldhA* mutant during biofilm growth, while no changes were detected in log phase cultures (Figure 6A,B). No changes were identified in the expression of *nhbA*, *mltB* or the minor pilin *pilX* under either condition (Figure 6A,B). To summarize, the expression of *pilE*, *siaD*, *nalP*, *mltA*, and *ampD* was downregulated during Δ*ldhA* biofilm formation, while *norB*, which is involved in anaerobic respiration, was upregulated. Furthermore, the expression of *narP* and *norB* was upregulated in the Δ*ldhA* mutant during log phase growth.

### Acetate Level Is Reduced in Δ*ldhA* Biofilms but Does Not Influence Biofilm Formation

**FIGURE 7:**
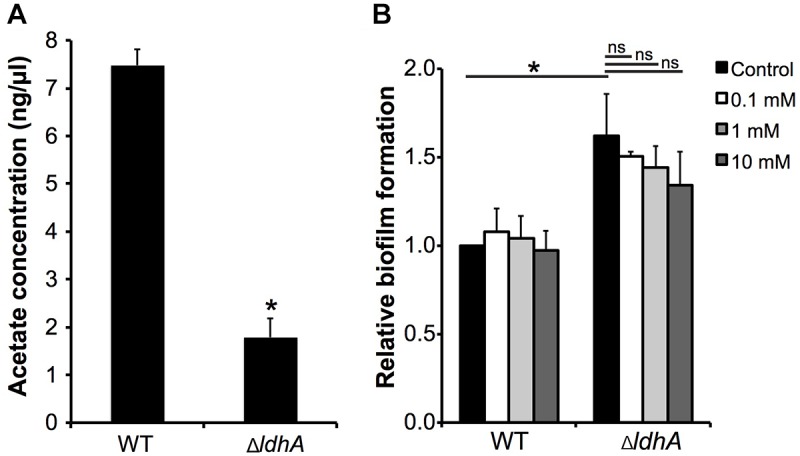
Acetate level is reduced in Δ*ldhA* biofilms but does not influence biofilm formation. **(A)** Quantification of acetate level in biofilm supernatants. Culture medium was collected from wild-type and Δ*ldhA* biofilms and concentration of acetate was quantified using Acetate Colorimetric Assay Kit. Experiment was performed three times in duplicate. **(B)** Biofilm formation by the wild-type and Δ*ldhA* in the presence of 0.1, 1 and 10 mM sodium acetate. Experiment was performed two times in triplicate. The bars represent the means, with error bars representing the standard deviation. ^∗^*p* < 0.05. ns, non-significant.

Accumulation of acetate and acetate intermediates are known to influence biofilm formation of several pathogens (Yang et al., 2005; Sharma-Kuinkel et al., 2009; Chen et al., 2015). We therefore speculated whether acetate played a role in biofilm formation of the Δ*ldhA* mutant. Supernatants were collected from 24 h old biofilms and the amount of acetate was quantified. We detected significant decrease in the amount of acetate in supernatants from Δ*ldhA* biofilms compared to that of the wild-type (Figure 7A). This indicated that reduction in the acetate levels might play a role in the increased biofilm formation by Δ*ldhA* mutant. To explore this possibility, we performed assays to measure biofilm formation by wild-type and Δ*ldhA* mutant in presence of 0.1, 1, and 10 mM sodium acetate. We did not detect significant reduction in biofilm formation by Δ*ldhA* mutant in presence of acetate (Figure 7B). This indicates that although there is a reduction in the level of acetate in Δ*ldhA* biofilm supernatants it does not play a role in the increase in biofilm formation.

## Discussion

In this study, we examined the role of lactate metabolism in meningococcal biofilm formation *in vitro*. We provide evidence that a D-lactate dehydrogenase, LdhA, has a negative impact on *N. meningitidis* biofilm formation. Loss of *ldhA* increased the level of bacterial aggregation and biofilm formation. This effect was associated with an increased autolytic rate and the amount of eDNA released.

Deletion of *ldhA* increased the level of biofilm formation. In meningococci, there is a direct relationship between surface hydrophobicity and biofilm formation (Yi et al., 2004). Our results show that the Δ*ldhA* mutant has increased surface hydrophobicity compared to that of the wild-type. Because the capsule affects bacterial hydrophobicity, we quantified the level of capsule. However, there was no difference in the amount of capsule between the wild-type and Δ*ldhA* mutant strains.

Treatment by DNase I abolished the enhanced aggregation and biofilm formation by Δ*ldhA*, suggesting that eDNA plays a role. It is well-known that eDNA plays a major role in biofilm formation in *N. meningitidis* and other pathogens that colonize the upper respiratory tract, such as *Pseudomonas aeruginosa*, *Streptococcus pneumoniae*, and *Haemophilus influenza* (Whitchurch et al., 2002; Jurcisek and Bakaletz, 2007; Hall-Stoodley et al., 2008; Lappann et al., 2010). The biofilm formation and architecture of meningococci exhibit strain-dependent variations. Biofilm formation by frequently carried meningococcal strains is dependent on eDNA, while hypervirulent strains of clonal complexes (cc) ST-8 and ST-11 do not depend on eDNA (Lappann et al., 2010). The level of biofilm formed by the wild-type FAM20 was not affected by the treatment of DNase I in our studies. Also, the treatment did not have an impact on Δ*cap* biofilm formation. This result is in agreement with the theory that strains such as FAM20, which belong to ST-11 cc, do not depend on eDNA. It is noteworthy that eDNA on streptococcal cell surfaces can increase hydrophobicity (Das et al., 2010). However, eDNA is also known to increase bacterial adhesion to surfaces (Rice et al., 2007; Das et al., 2010; Regina et al., 2014). Additionally, an enhanced aggregation can play a major role in bacterial adhesion to host cells (Mikaty et al., 2009; Kuwae et al., 2011; Engman et al., 2016). However, we did not detect differences in adhesion between the wild-type and Δ*ldhA* strains. The roles of eDNA in meningococcal adhesion and aggregation have not been well-studied, although its role is well established in biofilm formation (Lappann et al., 2010; Pérez-Ortega et al., 2017).

There is a link between eDNA-dependent biofilm formation and autolysis in meningococci (Lappann et al., 2010; Arenas and Tommassen, 2017). The Δ*ldhA* mutant exhibited an increased rate of autolysis, although the underlying mechanisms remain to be elucidated. Meningococcal autolysis can occur through the lytic transglycosylases MltA and MltB and the *N*-acetylmuramyl-L-alanine amidase AmpD (Lappann et al., 2010). Also, OMPLA is an important inducer of autolysis in meningococci during the stationary phase (Bos et al., 2005). DNA released during OMPLA-mediated autolysis plays a role in meningococcal biofilms and the ability to withstand shear forces and, therefore, contributes to long-term colonization (Lappann et al., 2010). However, ST-8 and ST-11 cc, including FAM20, lack a functional gene encoding OMPLA (Bos et al., 2005). Surprisingly, via RT-qPCR analysis, we detected reduced expression of *mltA* and *ampD*, which encode the known meningococcal autolysins in Δ*ldhA* biofilms. Studies have also shown that environmental factors, including pH, growth phase, and NaCl, can be responsible for bacterial autolysis (reviewed in Rice and Bayles, 2008).

We detected downregulation of the capsule biosynthesis gene *siaD* during Δ*ldhA* biofilm formation. Earlier studies have shown reductions in the transcripts of the capsule biosynthesis genes during biofilm formation (O’Dwyer et al., 2009). However, Neil et al. (2014) have reported biofilm formation by encapsulated meningococci upon adhesion to epithelial cells, with no reduction in capsule observed. Surprisingly, we detected downregulation of *pilE* in Δ*ldhA* within a biofilm. Deletion of *pilE* is known to abolish microcolony formation and reduce biofilm thickness, although these results were shown in a strain expressing class I Tfp (Lappann et al., 2006). FAM20 and all strains that belong to ST-8 and ST-11 cc form eDNA-independent biofilms and, express class II Tfp (Cehovin et al., 2010; Wormann et al., 2014). The Tfp is nonetheless important for the establishment of firm adhesion in the presence of mechanical forces (Mikaty et al., 2009). In our assays, we only evaluated the level of biofilm formation in a static environment. However, we did not detect any changes in *pilE* expression during the log growth phase. The attachment could, therefore, be established at earlier time points, and the *pilE* downregulation could occur upon biofilm mode of growth.

Reduced expression of *nalP* was detected during Δ*ldhA* biofilm growth. As mentioned earlier, the autotransporter NalP can influence the level of biofilm formation by cleaving cell-surface eDNA-binding molecules (Arenas et al., 2013). A decrease in the NalP level could potentially contribute to the increased eDNA-binding during biofilm formation in the Δ*ldhA* mutant. In the future, it would be interesting to study the role of NalP in increased biofilm formation in the ΔldhA mutant. A decrease in amount of NalP has been shown to correlate with increased meningococcal biofilm formation, however this might vary depending on the amount of eDNA present (Pérez-Ortega et al., 2017). We did not, however, detect any changes in *nalP* expression during log phase growth. The autotransporters AutA and AutB have also been linked to meningococcal biofilm formation (Arenas et al., 2015, 2016). However, since the *autA* gene in FAM20 (FAM18 derivative) is disrupted and the *autB* is out of frame they were excluded from the study (Peak et al., 1999; Ait-Tahar et al., 2000; van Ulsen et al., 2001).

Adaptations to oxygen limitations are important during the development of biofilms. The expression of *norB*, which is involved in anaerobic respiration, was induced in the Δ*ldhA* mutant both during log phase and biofilm growth. It has been reported that the expression of *norB*, which encodes the nitric oxide reductase NorB, increases in the presence of nitric oxide (Householder et al., 2000; Rock et al., 2007). NorB is important for growth in oxygen-deprived environments, and a lack of its activity has a drastic impact on biofilm formation in *N. gonorrhoeae* (Householder et al., 2000; Falsetta et al., 2009). Additionally, we detected upregulation of *narP* in Δ*ldhA* log phase cultures. NarP-deficiency reduces biofilm formation in meningococci (Jamet et al., 2013). However, we did not detect any differences in *narP* expression during biofilm growth in the Δ*ldhA* mutant.

The strain used in this study was of clonal complex ST-11, which is known to form poor biofilms independent of eDNA. It would be of interest to compare the expression of *ldhA* during growth in both carrier and virulent strains. However, it is highly unlikely that LdhA is directly involved in biofilm formation.

It is tempting to speculate that a metabolic shift due to the lack of *ldhA* rather than a direct effect is the cause of the observed phenotype. There is accumulating evidence that there is a correlation between energy metabolism and biofilm formation in pathogenic *Neisseria*. Both transcriptomic and proteomic analyses have revealed changes in metabolic enzymes that are important in pyruvate metabolism, glycolysis/gluconeogenesis, and the citric acid cycle during the biofilm mode of growth (Falsetta et al., 2009; O’Dwyer et al., 2009; van Alen et al., 2010; Phillips et al., 2012). In *Neisseria*, LdhA can both serve as D-LDH, converting D-lactate to pyruvate, and reversibly convert pyruvate to D-lactate as an NADH-pyruvate reductase. The metabolic shift upon *ldhA* deletion in *Neisseria* has not been studied extensively. However, it has been shown that D-lactate production is abolished (Atack et al., 2014). Deletion of *ldhA* in other pathogens, such as *E. coli* and *S. pneumoniae*, also results in the inability to produce D-lactate and an increase in acetate, pyruvate, and ethanol production from glucose. Acetate intermediates have been shown to play a role as metabolic signaling molecules in bacterial biofilms. In *E. coli*, the deletion of *ldhA* leads to the accumulation of acetyl-CoA, an intermediate in acetate metabolism, and increases the level of biofilm formation. Acetic acid has been shown to stimulate biofilm formation in *Staphylococcus aureus* and *Bacillus subtilis* by activating the expression of genes that encode proteins that are important in bacterial autolysis (Yang et al., 2005; Sharma-Kuinkel et al., 2009; Chen et al., 2015). To examine the possible role of acetate in Δ*ldhA* biofilm formation we measured the concentration of acetate in supernatants collected from biofilms. We detected significant reduction in acetate concentration. However when acetate level was raised we were not able to significantly reduce the amount of biofilm formed by the Δ*ldhA* mutant.

## Conclusion

In conclusion, we showed that a lack of *ldhA* in *N. meningitidis* increases surface hydrophobicity, bacterial aggregation, and biofilm formation. Based on our results, we suggest that the promoted aggregation and biofilm formation in the Δ*ldhA* mutant is dependent on an increase in autolysis-mediated eDNA release. It is not currently known whether *ldhA* mutation frequently occurs during colonization, and it would be interesting in the future to investigate *ldhA* expression during *in vivo* mimicking conditions, and further elucidate its possible role upon biofilm formation and meningococcal pathogenesis.

## Data Availability

All datasets generated for this study are included in the manuscript and/or the Supplementary Files.

## Author Contributions

SS, GW, FZ, and A-BJ conceived and designed the experiments, analyzed the data, and wrote the manuscript. SS, GW, FZ, and MA performed the experiments.

## Conflict of Interest Statement

The authors declare that the research was conducted in the absence of any commercial or financial relationships that could be construed as a potential conflict of interest.
