# Supplementary material for: Deletion of D-Lactate Dehydrogenase A in Neisseria meningitidis Promotes Biofilm Formation Through Increased Autolysis and Extracellular DNA Release
Source: Front Microbiol. 2019 Mar 5;10:422. doi: 10.3389/fmicb.2019.00422 (PMC6411758; doi:10.3389/fmicb.2019.00422)
Supplement: Supplementary file 1 [file Data_Sheet_1.PDF]

Supplementary Material

Deletion of D-lactate dehydrogenase A in *Neisseria meningitidis* promotes biofilm formation through increased autolysis and extracellular DNA release

Sara Sigurlásdóttir, Gabriela M. Wassing, Fanglei Zuo, Melanie Arts, and Ann-Beth Jonsson[#]

[#] Corresponding author: Ann-Beth Jonsson, E-mail: ann-beth.jonsson@su.se

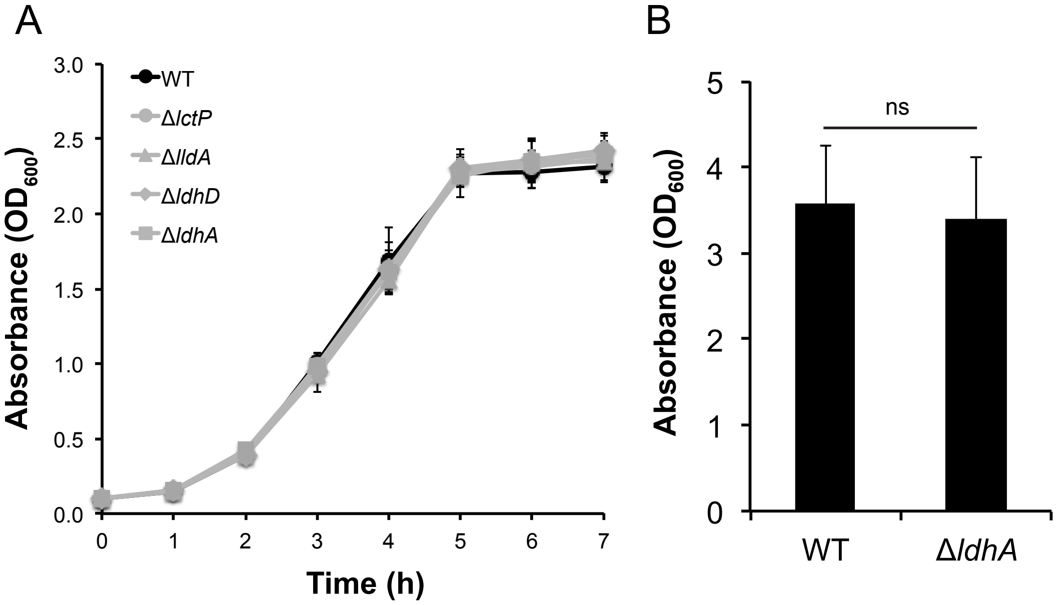

Supplementary Figure 1. Growth comparison between wild-type and mutant strains deficient in lactate metabolism. (A) Wild-type, Δ*lctP,* Δ*lldA,* Δ*ldhD,* and Δ*ldhA* bacteria were resuspended to an OD of 0.1 and samples were taken every hour for OD measurements. Experiments were performed three times. (B) Wild-type and Δ*ldhA* bacteria were resuspended to an OD of 0.05, and samples were taken after 24 h of growth in shaking conditions. Experiments were performed three times in duplicate. The bars represent the means, with error bars representing the standard deviations. ns, non-significant.

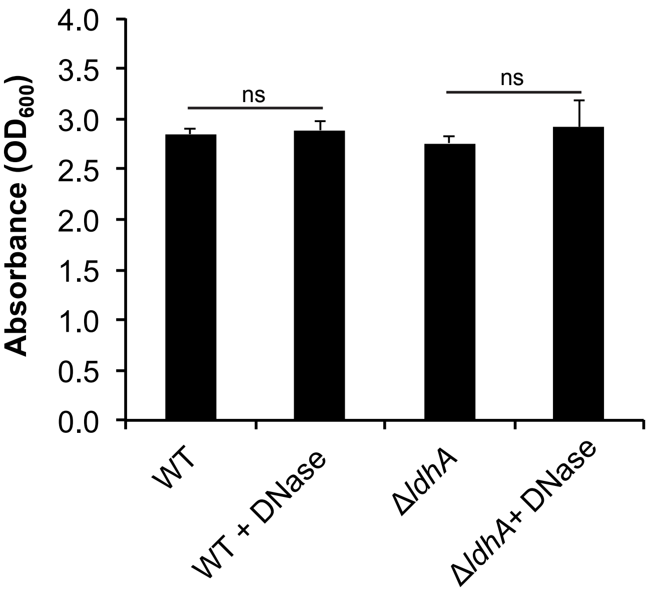

Supplementary Figure 2. DNase I does not affect the growth of meningococci. Wild-type and Δ*ldhA* bacteria were resuspended to an OD of 0.05 in the presence or absence of DNase I (100 µg/ml) and samples were taken after 24 h of growth in shaking conditions. Experiments were performed twice. The bars represent the means, with error bars representing the standard deviations. *$p < 0.05$. ns, non-significant.

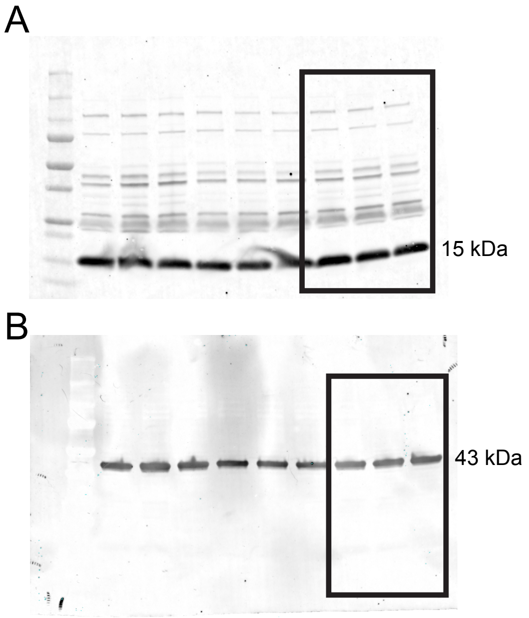

Supplementary Figure 3. Entire membrane from Figure 2B. (A) PilE detection. (B) EF-Tu detection.
